# Outcome prediction modelling for trauma patients: a German perspective

**DOI:** 10.1186/s13054-014-0616-8

**Published:** 2014-10-31

**Authors:** Omar Bouamra, Mehdi M Lesko

**Affiliations:** The Trauma Audit & Research Network, The University of Manchester, Salford Royal Hospital Trust, 3rd Floor Mayo Building, Salford, M6 8HD UK

## Abstract

Prognostic models may have some clinical advantages when predicting the outcome of individual trauma patients is relevant. The variables that are predicted to have a negative effect on outcome in a model can also guide clinicians in their resuscitation attempt of trauma victims.

This issue of *Critical Care* presents an article from Lefering and colleagues on modelling prediction of outcome for trauma patients [1]. This article is in fact an update of an existing model developed by the same authors.

Trauma registries have been established in several countries; the most important in terms of size are the National Trauma Database in the USA, the Trauma Audit and Research Network in England and, among many others, the TraumaRegister DGU in Germany. The interest in trauma has increased in the last few years, and it is predicted that trauma will be a top-three cause of death in the world by 2020. Trauma is already the leading cause of mortality in the under-40 age group. All of these facts have attracted interest from researchers to develop prognostic models to predict outcome for trauma patients. An important aspect of prognostic models is external validation, because most of the in-house models show good calibration and discrimination using their own data but lose a lot of their predictive power as soon as the models are tested on an external set of data, mainly because different countries/regions have a different case mix of the trauma population.

Lefering and colleagues conducted the derivation of the Revised Injury Severity Classification II model in a thorough way. The proposed model is an updated and improved version of a previous prediction model. Although the Injury Severity Score is used in most of the trauma-related prognostic models, the authors choose to ignore it – they rightly justified this because the Injury Severity Score has limitations such as disregarding multiple injuries in the same body region. Lefering and colleagues used the worst and the second-worst injury instead of the Injury Severity Score, which is the same approach used by Moore and colleagues [2] and Osler and colleagues [3]. It is well known that the Injury Severity Score overestimates mortality for higher values and underestimates mortality for lower values [4]. However, the authors’ approach is simple and avoids complexity.

Missing data are dealt with in this article using a subtle technique where the missing values are allocated to the reference category. Inclusion of cases with missing data gives more credibility to the prediction model.

As stated by Box and Draper, ‘Essentially, all models are wrong, but some are useful’ [5]. Prognostic models are useful when benchmarking trauma care between institutions using different outcome performance indicators. Despite the increasing number of trauma registries and the benchmarking models, the prognostic ability of these models hardly addresses individual patients in the clinical setting. There are two reasons for this. Firstly, there are concerns that the use of a model’s prediction may lead to a premature or inappropriate treatment withdrawal on the basis of a grim calculated probability. Secondly, the primary objective of these models is trauma care benchmarking and they are not meant to be used for prediction of individual patient outcome.

Despite these arguments, the models may still have some value for clinical purpose, especially in the case of multiple casualties with limited resources. In such scenarios, the limited resources may have to be allocated to those victims who are likely to benefit the most by having a better prognosis. Such scenarios are not uncommon when highly sophisticated trauma care is required, such as intensive care. This possible advantage of prognostic models has not so far been investigated. Furthermore, the correctable variables that have been included in the final models can have clinical implications, in that a trauma clinician may have to focus on correcting abnormal physiological indexes such as blood pressure, international normalised ratio or haemoglobin. In the same way, fluid resuscitation should perhaps also address the correction of acidosis (or base-deficit) alongside blood pressure.
